# Global, regional and national burden of diabetes mellitus type 2 attributable to low physical activity from 1990 to 2021 and projections to 2050: a finding from the global burden of disease study 2021

**DOI:** 10.3389/fcdhc.2025.1606330

**Published:** 2025-08-12

**Authors:** Ning Zhang, Xuan Qu, Lin Kang

**Affiliations:** Department of Geriatrics, Peking Union Medical College Hospital, Peking Union Medical College, Chinese Academy of Medical Sciences, Beijing, China

**Keywords:** global burden of disease, type 2 diabetes mellitus, low physical activity, epidemiological trends, projections

## Abstract

**Background:**

This study aims to assess the global burden of type 2 diabetes mellitus (T2DM) attributable to low physical activity from 1990 to 2021 and forecast of its global burden by 2050 using GBD 2021 data.

**Method:**

This study uses data from the GBD 2021 to examine the global burden of T2DM attributable to low physical activity, focusing on deaths, disability-adjusted life years (DALYs), years lived with disability (YLDs), and Years of Life Lost (YLLs). Descriptive analysis was performed across gender, age, region, and country for 1990 and 2021, using age-standardized rates. Trend analysis assessed the average changes in these rates from 1990 to 2021 by calculating the estimated annual percentage change (EAPC). Projections for future burden were made using the exponential smoothing (ES) model and the autoregressive integrated moving average (ARIMA) model.

**Result:**

In 2021, T2DM attributed to low physical activity caused 149,214 deaths and 5,523,050 DALYs, with significant increases since 1990. Both age-standardized mortality rate (ASMR) and age-standardized disability-adjusted life years (DALYs) rate (ASDR) rose, especially among females. The highest burden occurred in the 95+ and 70–74 age groups. High-SDI regions had the highest rates, with rapid increases in the high-middle SDI regions. Countries like the UAE, Montenegro, and Hungary showed the highest rates. Projections from 2022 to 2050 indicate a steady rise in deaths and DALYs, with a peak in 2050, though the rate of increase is slower according to the exponential smoothing model.

**Conclusion:**

The burden of T2DM attributable to low physical activity has steadily increased, with concerning future trends.

## 1 Introduction

Diabetes mellitus is a major global public health challenge, significantly contributing to mortality and disability-adjusted life years (DALYs) (1). The Global Burden of Disease (GBD) Study 2021 reports that the prevalence of diabetes has steadily increased over the past three decades, with projections indicating a continued rise until at least 2050 (1). Type 2 diabetes mellitus (T2DM) accounts for the majority of diabetes cases and is primarily driven by modifiable risk factors, including obesity, poor dietary habits, and physical inactivity (2). The global economic burden of T2DM is substantial, straining healthcare systems and disproportionately affecting low- and middle-income countries (LMICs) (3). Therefore, understanding the burden of T2DM and its associated risk factors is critical for developing effective prevention and management strategies.

Physical activity plays a pivotal role in the prevention and management of T2DM (4). Regular physical activity enhances insulin sensitivity, reduces inflammation, and improves glycemic control (5). Multiple epidemiological studies have demonstrated that higher levels of physical activity are associated with a lower incidence of T2DM and a reduced risk of diabetes-related complications (6–8). A meta-analysis found that individuals engaging in high levels of physical activity had a significantly lower risk of developing T2DM compared to those with sedentary lifestyles (9, 10). Furthermore, randomized controlled trials have shown that lifestyle interventions incorporating physical activity can reduce the incidence of T2DM by up to 58% in high-risk populations (11, 12). These findings underscore the urgent need for public health initiatives to promote physical activity and reduce sedentary behavior.

Despite the well-established benefits of physical activity, low physical activity remains a major contributor to the global burden of T2DM (13, 14). An analysis of GBD 2019 data estimated that 125,200 (95% UI: 62,100–208,350) T2DM-related deaths worldwide were attributable to low physical activity, marking a significant increase from 49,780 (95% UI: 24,520–84,620) in 1990 (14). Moreover, low physical activity is responsible for approximately 7% of the global T2DM burden, a proportion that continues to rise in parallel with increasing levels of physical inactivity worldwide (15). The economic consequences are substantial, with low physical activity-related deaths and healthcare costs exceeding $5 million and $67.5 billion annually, respectively (16, 17). Recognizing its profound impact on public health, the World Health Organization (WHO) has classified physical inactivity as the fourth leading risk factor for mortality over the past decade (18). However, a comprehensive, up-to-date analysis evaluating the impact of low physical activity on T2DM across different demographic and socioeconomic groups remains lacking.

The GBD 2021 data provides a systematic and comprehensive assessment of global epidemiological trends, quantifying the burden of 371 diseases and integrating 88 risk factors worldwide (19). In this study, we leverage GBD 2021 data to estimate the burden of T2DM attributable to low physical activity at global, regional, and national levels. Our analysis is stratified by sex, age group, and Sociodemographic Index (SDI) to identify disparities in disease burden. Furthermore, we project the impact of low physical activity on T2DM prevalence and DALYs from 2022 to 2050, providing critical insights for future health policy and intervention programs. Our findings will inform targeted strategies to promote physical activity as a key component of T2DM prevention and control. Addressing physical inactivity through policy measures, urban planning, and behavioral interventions could substantially mitigate the future burden of T2DM, ultimately improving global health outcomes.

## 2 Method

### 2.1 Data source

GBD 2021 study (https://vizhub.healthdata.org/gbd-results/) extensively collects and evaluates the latest global disease burden data on 371 diseases and injuries, and estimates the relationship between 88 risk factors and health outcomes (19). The data on T2DM deaths, disability-adjusted life-years (DALYs), years lived with disability (YLDs), and years of life lost (YLLs) attributable to low physical activity used in this study were all obtained from the GBD 2021 database. The GBD 2021 defined T2DM as fasting plasma glucose levels of ≥7 mmol/L or current hypoglycemic treatment, corresponding to ICD-10 codes E11-E11.1 and E11.3-E11.9. The primary data sources on low physical activity for the GBD study included the Global Physical Activity Questionnaire, the International Physical Activity Questionnaire, and various additional survey instruments (20).In the GBD study, the evaluation of physical activity for adults aged 25 and older focuses on durations of at least ten minutes across various life domains, including leisure and recreation, household and work activities, and transportation. The calculation of total metabolic equivalent-minutes per week is based on the frequency, duration, and intensity of the activities performed. The metabolic equivalent (MET) is defined as the ratio of the metabolic rate during activity to the metabolic rate at rest. One MET represents the energy expenditure associated with sitting quietly, approximately 1 kcal/kg/hour, or the oxygen consumption at rest, roughly 3.5 ml/kg/min. Activity levels are categorized into four groups based on the quartiles of total global MET-minutes per week: inactive (<600 MET-minutes weekly), low active (600–3999 MET-minutes weekly), moderately active (4000–7999 MET-minutes weekly), and highly active (≥8000 MET-minutes weekly) (20, 21). In the GBD 2021 analysis, the theoretical threshold for minimal risk associated with physical inactivity was established between 3600 and 4400 MET-minutes weekly. Therefore, less than 3600 MET-minutes per week was defined as low physical activity (20). The attribution of low physical activity to the T2DM burden followed GBD’s hierarchical risk framework, which necessitated multi-source evidence, biological plausibility, and dose-response relationships. Systematic reviews and meta-regressions estimated the relative risks of T2DM across low physical activity exposures, adjusted for confounding factors. Spatiotemporal Gaussian process regression synthesized global exposure data, with the TMREL set at 0 MET-min/week for complete inactivity. Attributable burden metrics (deaths, YLLs, YLDs, DALYs) were calculated using relative risks and exposure distributions, with the full methodology detailed in the GBD 2021 supplemental appendices (22).

### 2.2 Descriptive analysis

In this study, we examined the distribution characteristics of the burden of T2DM attributable to low physical activity globally and across different genders, age groups, regions and countries in 1990 and 2021. In GBD 2021 study, the formula for ASR calculation is as follows:


$$ASR=\frac{\sum_{i=1}^{A}a_{i}w_{i}}{\sum_{i=1}^{A}w_{i}}\times 100,000$$

Where i denotes the i th age group, $a_{i}$ represents age-specific rate, $w_{i}$ is the number of population (or weight) in the corresponding age groups of the selected reference standard population (23).

Uncertainty intervals (UIs) were constructed using the 2.5th and 97.5th percentiles from a 1000-draw distribution for each metric (24). Based on their SDI ratings, countries and territories in the GBD 2021 dataset are categorized into five groups: low (<0.46), low-middle (0.46-0.60), middle (0.61-0.69), high-middle (0.70-0.81), and high (>0.81) (25). All analyses were performed using R software (version 4.1.0). A *P*-value of less than 0.05 was considered statistically significant.

### 2.3 Trend analysis

The average trends in age-standardized mortality rate (ASMR), age-standardized DALYs rate (ASDR), age-standardized YLDs rate (ASYR) and age-standardized YLLs rate during 1990 to 2021 are assessed using the estimated annual percentage change (EAPC). The formula for calculating EAPC is as follows:


y=α+βx+ϵ

$$EAPC=\left(e^{\beta }-1\right)\times 100\%$$

Where y represents ln(ASR) , x denotes the calendar year and β is the slope obtained from the linear regression of the natural logarithm of the ASR on the year (26).

### 2.4 Forecasting analysis

In this study, the projections for the burden of T2DM attributable to low physical activity were performed using the ES model and the ARIMA model. The ARIMA model excels at identifying trends and seasonality within the data, whereas the ES model focuses on recent observations, offering a well-rounded perspective on potential future outcomes (27).

## 3 Result

### 3.1 Global burden of T2DM attributable to low physical activity

**Figure 1:**
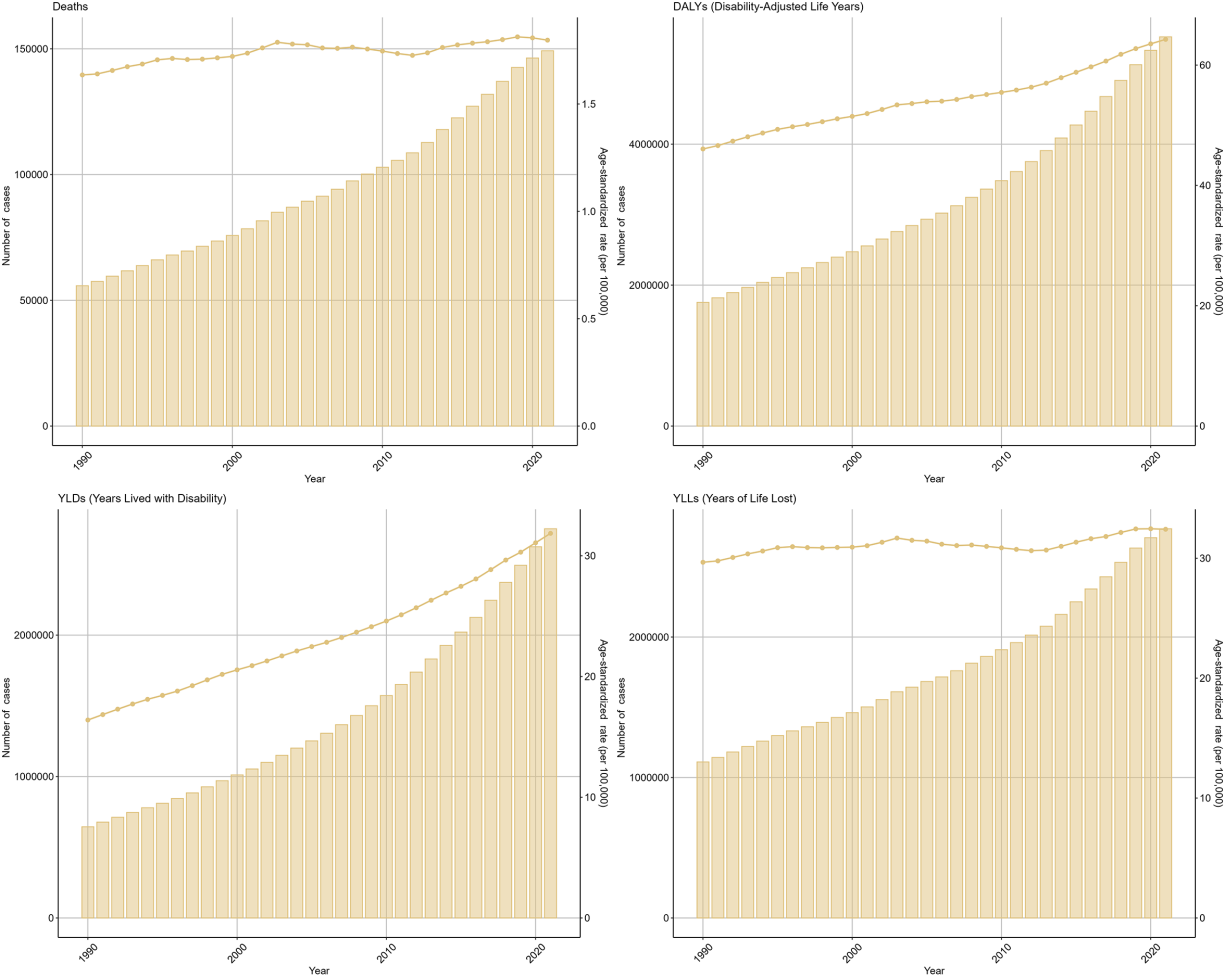
The global burden of type 2 diabetes mellitus attributable to low physical activity from 1990 to 2021.

In 2021, there were 149,214 (95% UI: 65,194-228,318) deaths due to T2DM attributable to low physical activity, an increase of 55,801 (95% UI: 24,049-85,577) in 1990. And the DALYs caused by T2DM attributable to low physical activity worldwide increased from 1755082 (95% UI: 756,859-2,694,173) in 1990 to 5,523,050 (95% UI: 2,407,128-8,638,535) in 2021. Additionally, the ASMR of T2DM attributable to low physical activity increased from 1.64 (95% UI: 0.71-2.51) in 1990 to 1.80 (95% UI: 0.79-2.75) in 2021, with an EAPC of 1.07 (95% *CI*: 0.70-1.44). While the ASDR increased from 46.06 (95% UI: 19.90-70.69) in 1990 to 64.27 (95% UI: 28.01-100.49) in 2021, with an EAPC of 1.67 (95% *CI*: 1.4-1.95) (**Figure 1**).

### 3.2 Sex-specific burden of T2DM attributable to low physical activity

In 2021, overall, the ASMR of T2DM attributable to low physical activity for females [2.03 (95% UI: 0.9-3.11)] was higher compared to that for males [1.50 (95% UI: 0.65-2.34)], similar pattern could be seen in the ASDR, the ASDR for females [74.38 (95% UI: 32.27-115.78)] was also higher than that for males [52.92 (95% UI: 22.89-83.1)] (**Supplementary Figure S1**). The deaths, DALYs, YLDs and YLLs of T2DM attributable to low physical activity stratified by gender is depicted in **Supplementary Figure S2**.

From 1990 to 2021, the ASMR of T2DM attributable to low physical activity for females increased from 1.89 (95% UI: 0.85-2.91) in 1990 to 2.03 (95% UI: 0.9-3.11) in 2021, with an EAPC of 0.18 (95% *CI*: 0.13-0.24). While the ASMR of T2DM attributable to low physical activity for males increased from 1.28 (95% UI: 0.53-1.95) in 1990 to 1.50 (95% UI: 0.65-2.34) in 2021, with an EAPC of 0.48 (95% *CI*: 0.42-0.54). Additionally, the ASDR of T2DM attributable to low physical activity for females increased from 53.94 (95% UI: 23.65-83.42) in 1990 to 74.38 (95% UI: 32.27-115.78) in 2021, with an EAPC of 0.95 (95% *CI*: 0.91-0.99). And the ASDR of T2DM attributable to low physical activity for males increased from 36.48 (95% UI: 15.55-56.64) in 1990 to 52.92 (95% UI: 22.89-83.1), with an EAPC of 1.08 (95% *CI*: 1.03-1.13) (**Supplementary Figure S3**). Trends in the deaths, DALYs, YLDs and YLLs of T2DM attributable to low physical activity stratified by gender are depicted in **Supplementary Figure S4**.

### 3.3 Age-specific burden of T2DM attributable to low physical activity

In 2021, the highest ASMR and ASDR of T2DM attributable to low physical activity occurred in 95+ age group [ASMR: 68.48 (95% UI: 28.87-107.35), [ASDR: 762.34 (95% UI: 328.25-1184.08)] (**Supplementary Figure S5**). The deaths, DALYs, YLDs and YLLs of T2DM attributable to low physical activity stratified by age is depicted in **Supplementary Figure S6**. From 1990 to 2021, the ASMR in the 80–84 age group increased the most, with an EAPC of 0.45 (95% *CI*: 0.39-0.52). Similarly, the highest growth in the ASDR occurred in the same age group (EAPC=0.95, 95% *CI*: 0.88-1.01) (**Supplementary Figure S7**). Trends in deaths, DALYs, YLDs and YLLs of T2DM attributable to low physical activity stratified by age is depicted in **Supplementary Figure S8**. Moreover, **Table 1** presents the number of deaths and the age-standardized mortality rate (ASMR) for T2DM attributable to low physical activity in 1990 and 2021, stratified by sex, age, and SDI region. **Table 2** provides the disability-adjusted life years (DALYs) and the age-standardized DALY rate (ASDR) for T2DM attributable to low physical activity in the same years, also stratified by sex, age, and SDI region.

**Table 1:** The deaths and age-standardized mortality rate (ASMR) of type 2 diabetes mellitus attributable to low physical activity in 1990 and 2021.

Category	1990		2021		EAPC (95% CI)
	Number (95% UI)	ASMR (95% UI)	Number (95% UI)	ASMR (95% UI)	
Global	55801 (24049-85577)	1.64 (0.71-2.51)	149214 (65194-228318)	1.8 (0.79-2.75)	1.07 (0.7-1.44)
Sex					
Female	37504 (16920-57636)	1.89 (0.85-2.91)	95108 (42059-145455)	2.03 (0.9-3.11)	0.18 (0.13-0.24)
Male	18297 (7497-27922)	1.28 (0.53-1.95)	54105 (23392-84094)	1.5 (0.65-2.34)	0.48 (0.42-0.54)
Age					
65–69 years	7473 (3307-11368)	6.05 (2.67-9.2)	17516 (7760-27393)	6.35 (2.81-9.93)	-0.02 (-0.12-0.08)
70–74 years	8561 (3627-13172)	10.11 (4.28-15.56)	22246 (9465-34315)	10.81 (4.6-16.67)	0.15 (0.1-0.2)
75–79 years	10157 (4400-15323)	16.5 (7.15-24.89)	24210 (10243-36944)	18.36 (7.77-28.01)	0.27 (0.22-0.32)
80–84 years	9365 (4110-14370)	26.47 (11.62-40.62)	26301 (11506-40090)	30.03 (13.14-45.77)	0.45 (0.39-0.52)
85–89 years	5515 (2398-8518)	36.49 (15.87-56.37)	19029 (8129-29585)	41.62 (17.78-64.71)	0.5 (0.38-0.63)
90–94 years	2195 (955-3394)	51.23 (22.3-79.21)	10060 (4327-15556)	56.23 (24.19-86.96)	0.41 (0.26-0.55)
95+ years	675 (287-1058)	66.26 (28.22-103.96)	3732 (1573-5851)	68.48 (28.87-107.35)	0.24 (0.11-0.37)
SDI region					
Low SDI	3512 (1430-5471)	2.04 (0.83-3.19)	8886 (3673-14049)	2.33 (0.96-3.7)	0.48 (0.37-0.58)
Low-middle SDI	10383 (4421-15795)	2.19 (0.93-3.34)	37852 (16402-58173)	3.22 (1.39-4.96)	1.38 (1.31-1.45)
Middle SDI	16217 (6985-24854)	2.03 (0.87-3.13)	55677 (24646-84455)	2.32 (1.03-3.53)	0.42 (0.36-0.47)
High-middle SDI	10833 (4664-16594)	1.28 (0.56-1.95)	25209 (11305-38576)	1.3 (0.58-1.98)	0.05 (-0.03-0.13)
High SDI	14763 (6325-22565)	1.31 (0.56-2.02)	21403 (8958-33305)	0.88 (0.37-1.37)	-1.64 (-1.84–1.44)

ASMR, Age-standardized mortality rate per 100,000 population; EAPC, Estimated annual percentage change; UI, Uncertainty interval; CI, Confidence interval.

**Table 2:** The DALYs and age-standardized DALYs rate (ASDR) of type 2 diabetes mellitus attributable to low physical activity in 1990 and 2021.

Category	1990		2021		EAPC (95% *CI*)
	DALYs Number (95% UI)	ASDR (95% UI)	DALYs Number (95% UI)	ASDR (95% UI)	
Global	1755082 (756859-2694173)	46.06 (19.9-70.69)	5523050 (2407128-8638535)	64.27 (28.01-100.49)	1.67 (1.4-1.95)
Sex					
Female	1133445 (495920-1751492)	53.94 (23.65-83.42)	3428400 (1487012-5330556)	74.38 (32.27-115.78)	0.95 (0.91-0.99)
Male	621637 (264577-973518)	36.48 (15.55-56.64)	2094650 (907065-3290688)	52.92 (22.89-83.1)	1.08 (1.03-1.13)
Age					
65–69 years	281079 (125847-433897)	227.39 (101.81-351.02)	843872 (363920-1323560)	305.93 (131.93-479.83)	0.82 (0.76-0.89)
70–74 years	259958 (110666-410902)	307.06 (130.72-485.35)	849375 (378643-1335835)	412.64 (183.95-648.97)	0.91 (0.87-0.94)
75–79 years	237680 (103763-370776)	386.12 (168.57-602.34)	687831 (296455-1088606)	521.54 (224.78-825.42)	0.88 (0.84-0.92)
80–84 years	169794 (73886-262385)	479.97 (208.86-741.7)	559468 (247727-869138)	638.79 (282.85-992.36)	0.95 (0.88-1.01)
85–89 years	76152 (32832-118421)	503.95 (217.27-783.67)	302969 (133983-472436)	662.64 (293.04-1033.28)	0.94 (0.84-1.05)
90–94 years	24245 (10403-38055)	565.8 (242.76-888.05)	128090 (55632-198193)	716.01 (310.98-1107.88)	0.84 (0.72-0.97)
95+ years	6498 (2763-10193)	638.22 (271.39-1001.19)	41550 (17890-64536)	762.34 (328.25-1184.08)	0.66 (0.56-0.76)
SDI region					
Low SDI	103907 (43162-161519)	50.22 (20.71-78.34)	295801 (125367-470146)	62.74 (26.4-99.23)	0.68 (0.61-0.74)
Low-middle SDI	318972 (136875-490980)	56.9 (24.41-87.64)	1204356 (519769-1871158)	88.59 (38.29-137.73)	1.46 (1.41-1.5)
Middle SDI	548693 (237748-847190)	56.85 (24.6-88.05)	2007029 (877454-3117402)	75.81 (33.18-117.39)	0.82 (0.77-0.86)
High-middle SDI	348797 (151116-533856)	36.66 (15.98-56.34)	950867 (416024-1492242)	48.14 (21.04-75.47)	0.79 (0.74-0.85)
High SDI	431738 (187455-669806)	38.79 (16.84-60.23)	1057953 (470182-1697065)	52.1 (23.03-84.44)	0.74 (0.61-0.86)

ASDR, Age-standardized DALYs rate per 100,000 population; EAPC, Estimated annual percentage change; UI, Uncertainty interval; CI, Confidence interval.

### 3.4 Regional and national burden of T2DM attributable to low physical activity

In 2021, the highest ASMR of T2DM attributable to low physical activity occurred in Low-middle SDI region [3.22 (95% UI: 1.39-4.96)]. Similarly, the highest ASDR occurred in the same region [88.59 (95% UI: 38.29-137.73)] (**Supplementary Figure S9**). The deaths, DALYs, YLDs and YLLs of T2DM attributable to low physical activity stratified by SDI region is depicted in **Supplementary Figure S10**. From 1990 to 2021, the highest growth in the ASMR of T2DM attributable to low physical activity occurred in Low-middle SDI region (EAPC=1.38, 95% *CI*: 1.31-1.45). Similarly, the highest growth in the ASDR occurred in the same region (EAPC=1.46, 95% *CI*: 1.41-1.50) (**Supplementary Figure S11**). Trends in the deaths, DALYs, YLDs and YLLs of T2DM attributable to low physical activity stratified by SDI region are depicted in **Supplementary Figure S12**.

**Figure 2:**
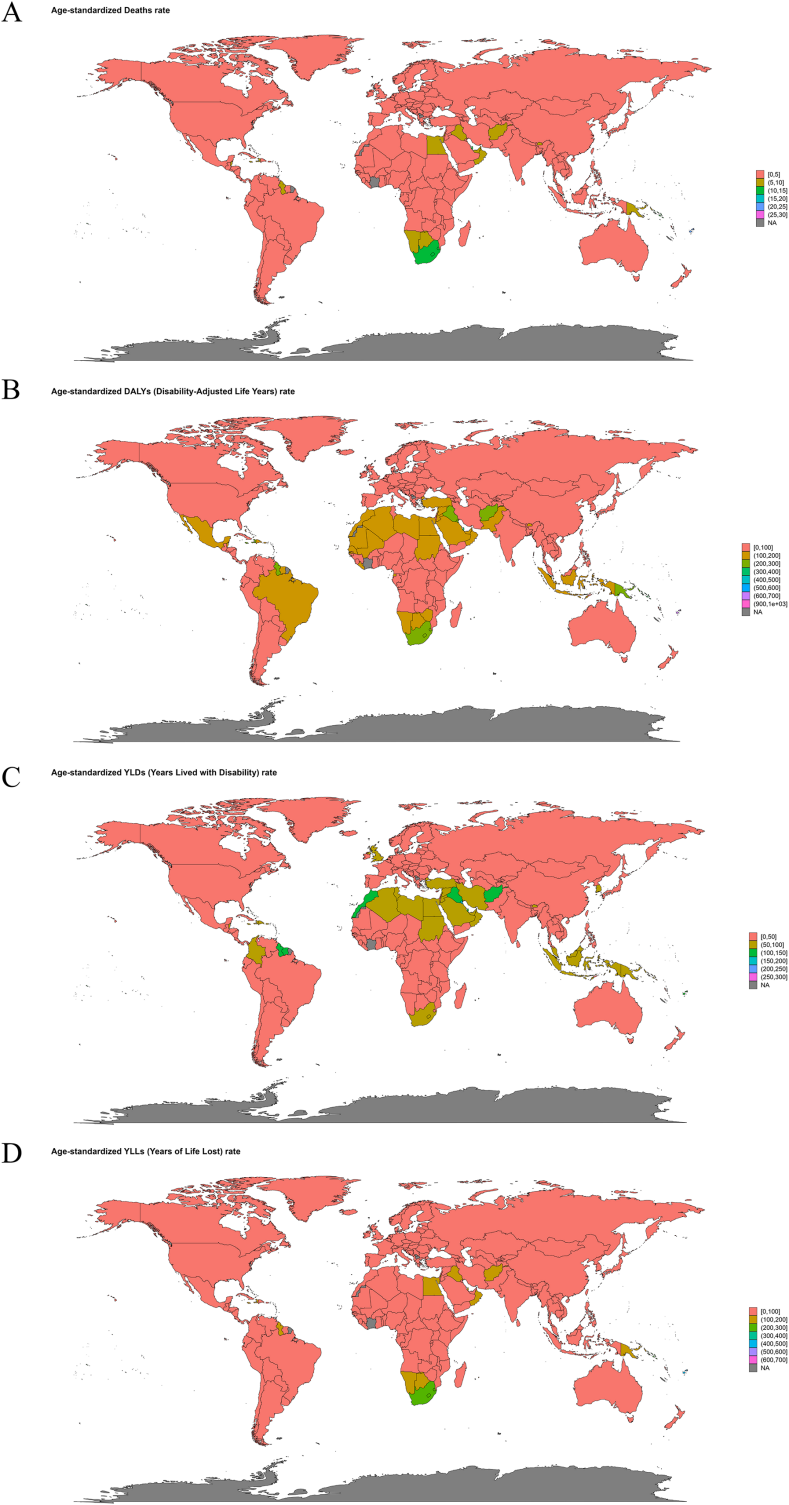
World maps showing age-standardized rates of type 2 diabetes mellitus attributable to low physical activity by country in 2021: **(A)** Age-standardized death rates (per 100,000 population); **(B)** Age-standardized Disability-Adjusted Life Years (DALYs) rates (per 100,000 population); **(C)** Age-standardized Years Lived with Disability (YLDs) rates (per 100,000 population); **(D)** Age-standardized Years of Life Lost (YLLs) rates (per 100,000 population). Each map uses color gradients to represent rate ranges, with red indicating higher burden and blue lower burden. Data derived from the Global Burden of Disease Study 2021.

**Figure 3:**
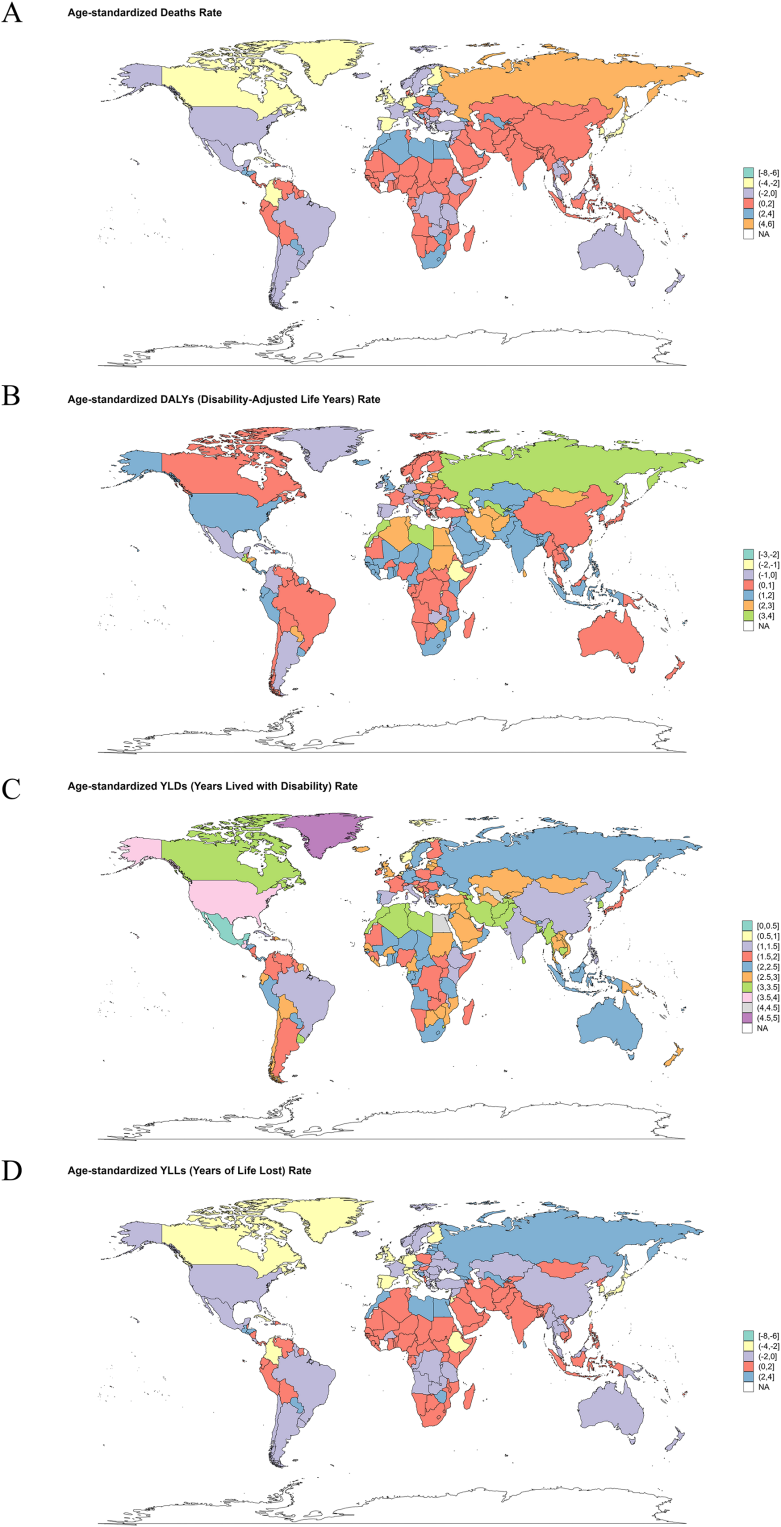
World map for the estimated annual percentage change (EAPC) of type 2 diabetes mellitus burden attributable to low physical activity from 1990 to 2021: **(A)** EAPC of age-standardized mortality rate (ASMR); **(B)** EAPC of age-standardized Disability-Adjusted Life Years (DALYs) rate (ASDR); **(C)** EAPC of age-standardized Years Lived with Disability (YLDs) rate (ASYR); **(D)** EAPC of age-standardized Years of Life Lost (YLLs) rate.

In 2021, the three countries with highest ASMR of T2DM attributable to low physical activity were Marshall Islands [27.56 (95% UI: 10.76-45.29)], Fiji [22.83 (95% UI: 10.12-38.75)] and Kiribati [22.20 (95% UI: 9.08-37.1)], while the three countries with highest ASDR were Marshall Islands [943.68 (95% UI: 406.21-1461.59)], Kiribati [649.74 (95% UI: 274.52-1059.85)] and Federated States of Micronesia [635.85 (95% UI: 283.56-980.43)] (**Figure 2** **).** The deaths, DALYs, YLDs and YLLs of T2DM attributable to low physical activity stratified by country is depicted in **Supplementary Figure S13**. From 1990 to 2021, the three countries with the fastest increase in ASMR of T2DM attributable to low physical activity are Mauritius (EAPC=5.64, 95% *CI*: 4.82-6.46), Russian Federation (EAPC=5.27, 95% *CI*: 3.94-6.61), Bosnia and Herzegovina (EAPC=5.04, 95% *CI*: 4.39-5.69). And the three countries with the fastest increase in ASDR of T2DM attributable to low physical activity are Mauritius (EAPC=4.88, 95% *CI*: 4.27-5.50), Guatemala (EAPC=4.39, 95% *CI*: 3.23-5.57), Bosnia and Herzegovina (EAPC=4.27, 95% *CI*: 3.66-4.89) (**Figure 3**).

### 3.5 Projection to 2050

**Figure 4:**
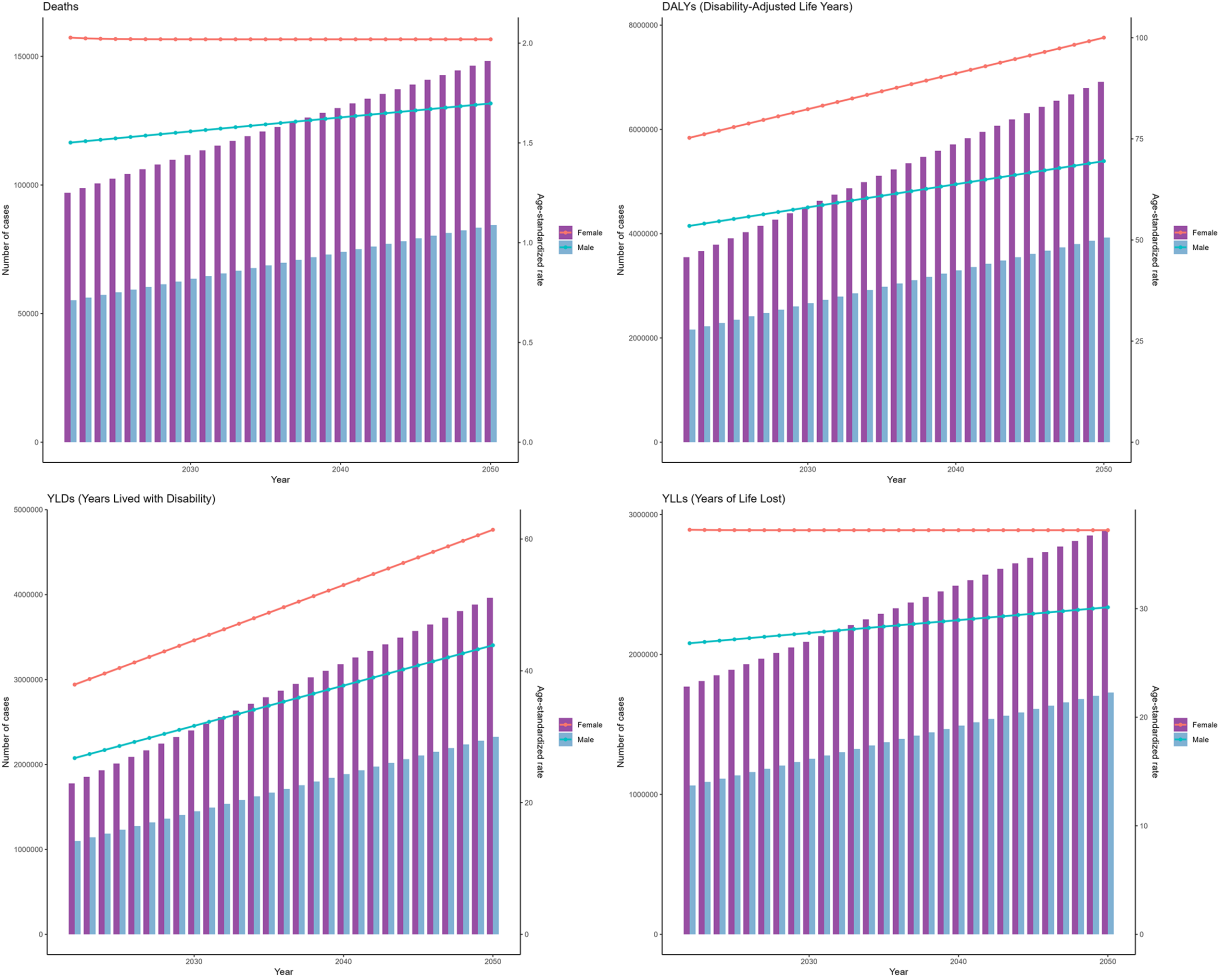
Projections to 2050 of the global burden of type 2 diabetes mellitus attributable to low physical activity performed using the autoregressive integrated moving average (ARIMA) model.

As projected by the ARIMA model, between 2022 and 2050, the deaths, DALYs, YLDs, and YLLs for T2DM attributable to low physical activity are projected to increase annually for both males and females. Additionally, the ASMR of T2DM attributable to low physical activity is anticipated to increase linearly for males from 2022 to 2050, while to remain stable for females (**Figure 4**). The ASDR of T2DM attributable to low physical activity are anticipated to increase linearly both for females and males from 2022 to 2050 (**Figure 4**).

**Figure 5:**
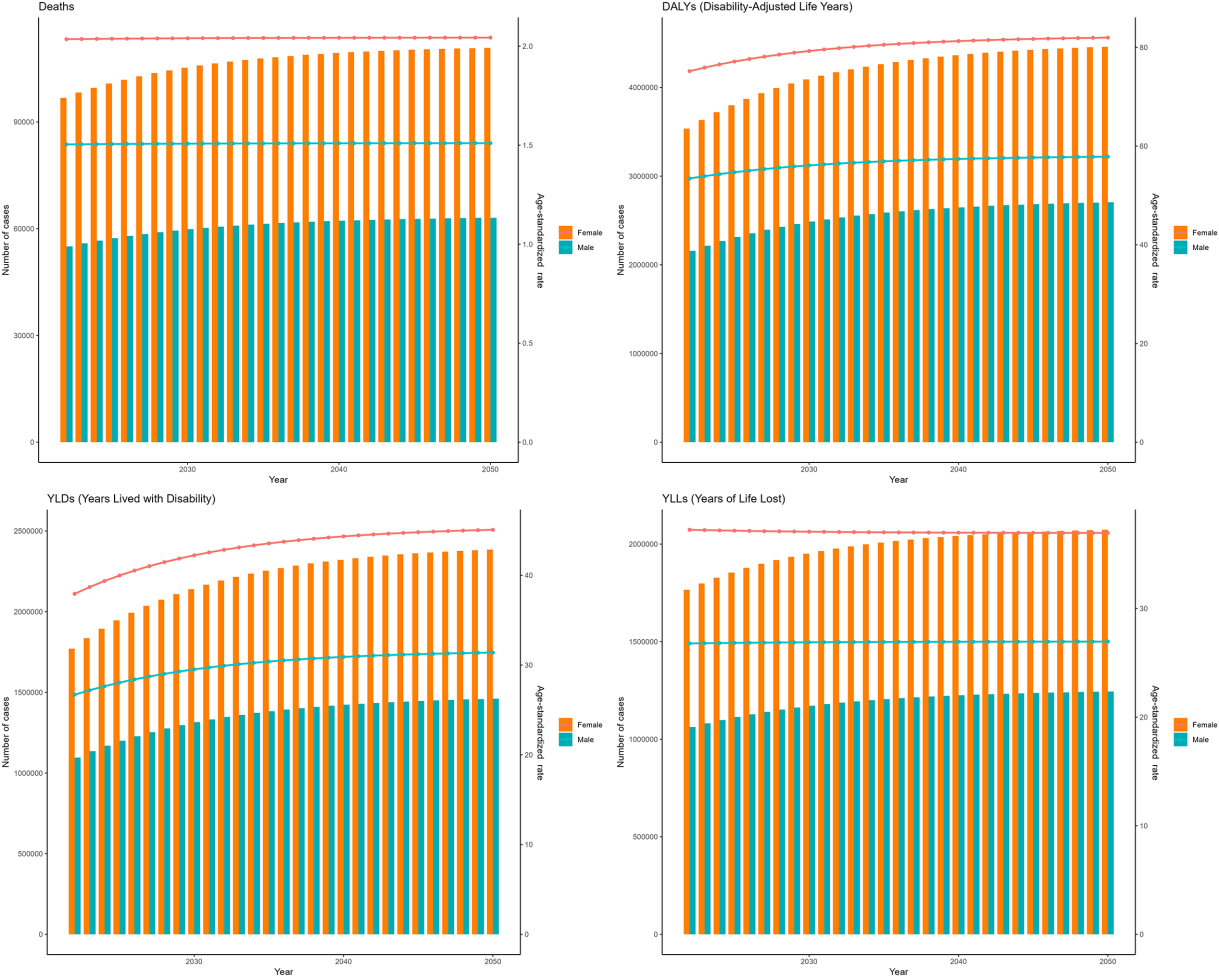
Projections to 2050 of the global burden of type 2 diabetes mellitus attributable to low physical activity performed using the exponential smoothing (ES) model.

As projected by the ES model, between 2022 and 2050, the deaths, DALYs, and YLDs, YLLs for T2DM attributable to low physical activity are projected to increase annually for both males and females. However, unlike the predictions of the ARIMA model, the growth rates of ASMR and ASDR are expected to slow down year by year (**Figure 5**).

## 4 Discussion

The findings of this study provide a comprehensive assessment of the global burden of T2DM attributable to low physical activity from 1990 to 2021, with projections extending to 2050. Utilizing data from the GBD 2021 study, our analysis highlights the increasing mortality and DALYs associated with low physical activity-related T2DM, alongside persistent disparities across sex, age groups, and geographic regions. The findings indicate that T2DM-related deaths and DALYs due to low physical activity continued to rise in 2021, with projections suggesting a sustained upward trajectory, particularly in high-income and certain middle-income countries. Sex- and age-stratified analyses reveal that women experience a higher low physical activity-related T2DM burden than men, particularly among elderly populations. At the country level, the United Arab Emirates, Montenegro, and Hungary exhibit the highest low physical activity-related T2DM burden, whereas Mauritius, Russia, and Bosnia and Herzegovina show the fastest growth rates. These findings underscore the continued rise in the global T2DM burden and the substantial heterogeneity in low physical activity’s impact across populations, emphasizing the need for targeted, population-specific interventions to mitigate future disease burden.

A striking finding is the disproportionate burden among females, who exhibited higher age-standardized mortality rates (ASMR: 2.03 *vs*. 1.50 per 100,000) and age-standardized disability-adjusted life year rates (ASDR: 74.38 *vs*. 52.92 per 100,000) compared to males. This disparity may stem from a combination of biological and sociocultural factors. Biologically, hormonal influences on insulin resistance, particularly postmenopausal declines in estrogen, may contribute to heightened T2DM susceptibility (28, 29). Socioculturally, women in many regions face barriers to physical activity due to caregiving responsibilities, safety concerns, and limited access to recreational facilities (30, 31). Additionally, postmenopausal women experience accelerated declines in muscle mass and metabolic health, further amplifying their risk of T2DM (32, 33). These findings highlight the urgent need for gender-sensitive interventions that address structural inequities and promote safe, accessible opportunities for physical activity. The highest burden of low physical activity-related T2DM was observed in the ≥95-year age group (ASMR: 68.48 per 100,000; ASDR: 762.34 per 100,000), with substantial increases beginning in the 80–84-year age cohort. Aging populations face compounded risks due to sarcopenia, chronic inflammation, and multimorbidity, all of which impair glucose regulation (34, 35). Moreover, older adults frequently encounter mobility limitations that restrict their ability to engage in moderate-to-vigorous physical activity (36, 37). This demographic shift underscores the critical need for age-tailored interventions, such as resistance training and community-based programs, to mitigate functional decline and enhance metabolic health in older adults.

We found that high-SDI regions exhibited the highest absolute burden of low physical activity-related T2DM, likely driven by sedentary occupations, car dependency, and high-calorie diets. In contrast, the most rapid increases in ASMR and ASDR were observed in low-middle SDI regions (EAPC: 1.38-1.46), where urbanization has disrupted traditional active lifestyles, and economic transitions have led to greater access to processed foods and screen-based entertainment (38, 39). Countries such as Mauritius and Bosnia and Herzegovina, which have the fastest-growing burdens, exemplify the intersection of Westernized lifestyles with inadequate public health infrastructure (40). Conversely, while high-SDI nations benefit from well-established healthcare systems, they require stronger policies to counteract entrenched sedentary behaviors. Small island nations, including the Marshall Islands and Kiribati, reported the highest ASMR and ASDR in 2021. These populations face unique challenges, such as limited land for physical activity, heavy reliance on imported energy-dense foods, and high obesity rates (41, 42). Historical colonial influences and economic dependencies further exacerbate these issues, underscoring the need for culturally adapted interventions. For instance, integrating traditional activities such as fishing or communal farming into structured physical activity programs could enhance community engagement and feasibility. Moreover, limited medical resources and inadequate health education contribute to low levels of T2DM prevention and management in low-SDI regions, further exacerbating disease severity. These factors collectively increase the risk of low physical activity-related T2DM among older adults in low-SDI areas, leading to heightened morbidity and mortality. Addressing these disparities requires tailored public health strategies that prioritize accessible and culturally relevant physical activity interventions.

Our study reveals that in 2021, low physical activity contributed to 149,214 deaths and 5.5 million DALYs globally, representing a significant increase since 1990. The ASMR and ASDR rose annually by 1.07% and 1.67%, respectively. These trends mirror broader epidemiological shifts in non-communicable diseases (NCDs), where urbanization, sedentary lifestyles, and obesogenic environments have exacerbated metabolic disorders (43). Our projection models (ARIMA and ES) further anticipate a continued rise in low physical activity-attributable deaths and DALYs through 2050, albeit with a potential deceleration in growth rates. These findings align with previous GBD analyses on obesity and T2DM (44, 45), reinforcing the urgency of sustained preventive measures.

Our findings demonstrate significant increases in YLDs attributable to low physical activity-induced T2DM from 1990 to 2021. The substantial burden of YLDs highlights that T2DM not only causes premature mortality but also leads to prolonged periods of disability, particularly among female patients and the oldest age groups (≥95 years). This gender disparity may reflect biological differences in diabetes complications as well as sociocultural factors that affect healthcare utilization. The age-related pattern suggests that accumulated disease duration and multimorbidity significantly contribute to the disability burden in elderly populations.

The observed patterns of YLLs indicate that low physical activity-related T2DM significantly contributes to premature mortality on a global scale. Although absolute YLLs are highest in high-SDI regions, the most rapid increase is occurring in low-to-middle SDI countries. This suggests the emergence of an epidemic in these regions, where healthcare systems may be ill-prepared to address the dual burden of communicable and non-communicable diseases. Furthermore, the relatively smaller gender gap in YLLs compared to YLDs indicates that while women experience greater disability, the mortality risks associated with T2DM are more evenly distributed between sexes.

Although individuals with diabetes generally face an increased risk of mortality compared to non-diabetic individuals, evidence suggests that physical activity plays a crucial role in mitigating or even offsetting this excess risk (46). Insufficient physical activity contributes to reduced cardiorespiratory fitness, a key factor influencing the onset, progression, and prognosis of T2DM. This decline is strongly correlated with heightened cardiovascular mortality, all-cause mortality, and the risk of multiple cancers (10, 47, 48). Mechanistically, low physical activity exacerbates insulin resistance by impairing glucose uptake and utilization in skeletal muscle (49). Additionally, low physical activity alters adipose tissue distribution and function, leading to increased abdominal fat accumulation—an established driver of insulin resistance (50). Importantly, any form of physical activity can improve metabolic health by enhancing pancreatic beta-cell function, regulating vascular function, and reducing systemic inflammation—all of which contribute to better disease management and lower health risks (5, 6). Furthermore, evidence suggests that substituting sedentary behavior or light physical activity with moderate-to-vigorous physical activity may also improve sleep quality, which is beneficial for glycemic control and T2DM management (51). In summary, physical activity is essential for maintaining normal glucose metabolism, while low physical activity exacerbates metabolic dysfunction and increases the risk of mortality in individuals with diabetes.

While our study provides critical insights, several limitations warrant consideration. First, GBD estimates rely on modeled data, which may underestimate the true impact of low physical activity due to incomplete surveillance, particularly in low-resource settings. Second, attributing T2DM burden to low physical activity is inherently complex, as it involves interactions with multiple risk factors (e.g., diet, genetics), and residual confounding may obscure these relationships. Third, our projections assume linear trends in risk factor exposure, which may not fully account for the impact of disruptive events such as pandemics, economic crises, or shifts in health policies. Furthermore, while type 1 diabetes and other diabetes subtypes also contribute significantly to the global disease burden, our analysis focused exclusively on T2DM due to data limitations. Future research should: Investigate the synergistic effects of low physical activity with emerging risk factors, such as air pollution and psychosocial stress. Evaluate the cost-effectiveness of large-scale physical activity interventions using real-world data.

## 5 Conclusion

The global burden of T2DM attributable to low physical activity represents a preventable public health crisis with profound health, economic, and social consequences. Our findings underscore the urgent need for equitable, scalable, and innovative strategies to combat sedentary lifestyles across all populations. To translate our recommendations into practice, specific strategies and interventions at the clinical, community, and policy levels are essential. At the clinical level, healthcare providers should routinely screen patients for physical activity levels and offer tailored exercise prescriptions, which have been shown to effectively reduce the risk of T2DM and its complications (52). Integrating physical activity counseling into routine care can empower patients to adopt and maintain an active lifestyle. At the community level, establishing accessible and affordable recreational facilities, such as community centers, parks, and walking trails, can foster an environment conducive to physical activity (53). Furthermore, community-based interventions, such as walking groups and exercise classes, can provide social support and motivation, encouraging sustained participation. At the policy level, governments should implement policies that support physical activity, such as mandating safe cycling and walking infrastructure, promoting active transportation, and providing incentives for employers to support workplace physical activity programs. These multi-level interventions, when implemented in concert, have the potential to mitigate the projected rise in the burden of T2DM, fostering healthier populations and more sustainable healthcare systems.

## Data Availability

The original contributions presented in the study are included in the article/**Supplementary Material**. Further inquiries can be directed to the corresponding author.
