# Supplementary material for: Global, regional and national burden of diabetes mellitus type 2 attributable to low physical activity from 1990 to 2021 and projections to 2050: a finding from the global burden of disease study 2021
Source: Front Clin Diabetes Healthc. 2025 Aug 12;6:1606330. doi: 10.3389/fcdhc.2025.1606330 (PMC12379646; doi:10.3389/fcdhc.2025.1606330)
Supplement: Supplementary file 1 [file DataSheet1.docx]

**Supplemental Figure legend**

**Figure S1** The age-standardized mortality rate (ASMR), age-standardized DALYs rate (ASDR), age-standardized YLDs rate (ASYR) and age-standardized YLLs rate of diabetes mellitus type 2 attributable to low physical activity by sex in 2021.

**Figure S2** The deaths, DALYs, YLDs and YLLs of diabetes mellitus type 2 attributable to low physical activity by sex in 2021.

**Figure S3** Trends in the age-standardized mortality rate (ASMR), age-standardized DALYs rate (ASDR), age-standardized YLDs rate (ASYR) and age-standardized YLLs rate of diabetes mellitus type 2 attributable to low physical activity by sex from 1990 to 2021.

**Figure S4** Trends in the deaths, DALYs, YLDs and YLLs of diabetes mellitus type 2 attributable to low physical activity) by sex from 1990 to 2021.

**Figure S5** The age-standardized mortality rate (ASMR), age-standardized DALYs rate (ASDR), age-standardized YLDs rate (ASYR) and age-standardized YLLs rate of diabetes mellitus type 2 attributable to low physical activity by age in 2021.

**Figure S6** The deaths, DALYs, YLDs and YLLs of diabetes mellitus type 2 attributable to low physical activity by age in 2021.

**Figure S7** Trends in the age-standardized mortality rate (ASMR), age-standardized DALYs rate (ASDR), age-standardized YLDs rate (ASYR) and age-standardized YLLs rate of diabetes mellitus type 2 attributable to low physical activity by age from 1990 to 2021.

**Figure S8** Trends in the deaths, DALYs, YLDs and YLLs of diabetes mellitus type 2 attributable to low physical activity by age from 1990 to 2021.

**Figure S9** The age-standardized mortality rate (ASMR), age-standardized DALYs rate (ASDR), age-standardized YLDs rate (ASYR) and age-standardized YLLs rate of diabetes mellitus type 2 attributable to low physical activity by SDI region in 2021.

**Figure S10** The deaths, DALYs, YLDs and YLLs of diabetes mellitus type 2 attributable to low physical activity by SDI region in 2021.

**Figure S11** Trends in the age-standardized mortality rate (ASMR), age-standardized DALYs rate (ASDR), age-standardized YLDs rate (ASYR) and age-standardized YLLs rate of diabetes mellitus type 2 attributable to low physical activity by SDI region from 1990 to 2021.

**Figure S12** Trends in the deaths, DALYs, YLDs and YLLs of diabetes mellitus type 2 attributable to low physical activity by SDI region from 1990 to 2021.

**Figure S13** World map of the deaths, DALYs, YLDs and YLLs of diabetes mellitus type 2 attributable to low physical activity in 2021.
