# Supplementary figures and images for: Global, regional and national burden of diabetes mellitus type 2 attributable to low physical activity from 1990 to 2021 and projections to 2050: a finding from the global burden of disease study 2021
Source: Front Clin Diabetes Healthc. 2025 Aug 12;6:1606330. doi: 10.3389/fcdhc.2025.1606330 (PMC12379646; doi:10.3389/fcdhc.2025.1606330)

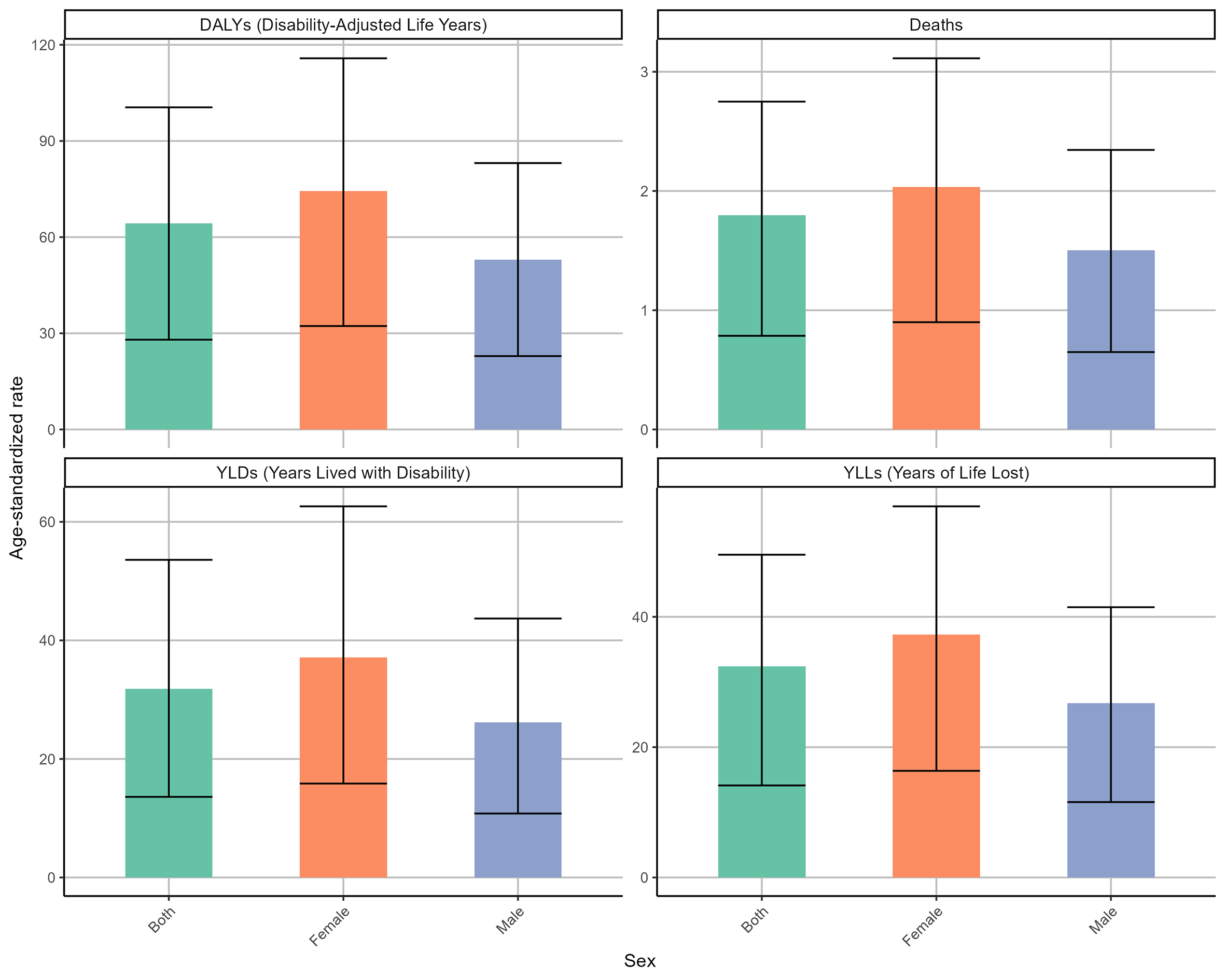

Supplement: Supplementary file 2 [file Image1.tif]

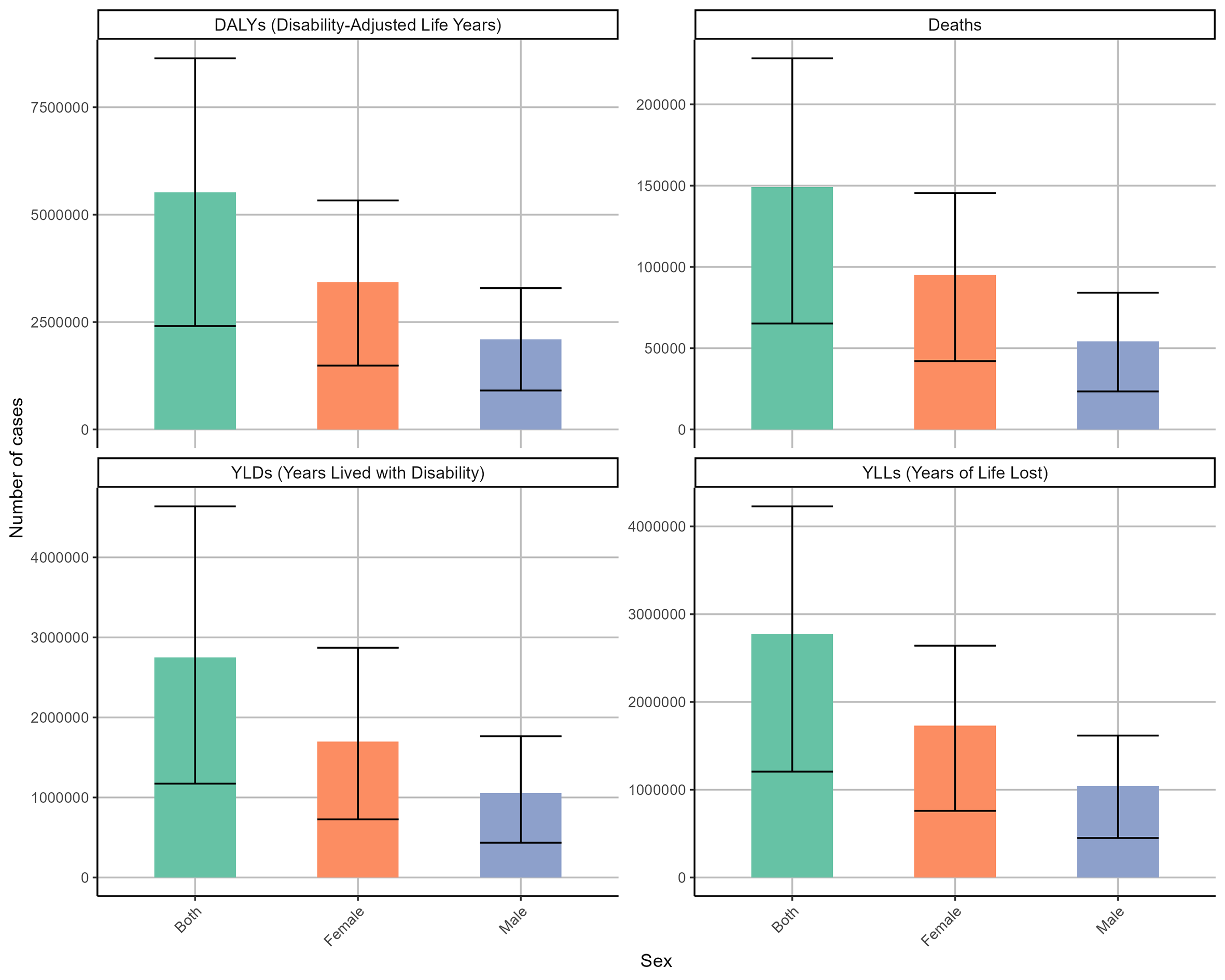

Supplement: Supplementary file 3 [file Image2.tif]

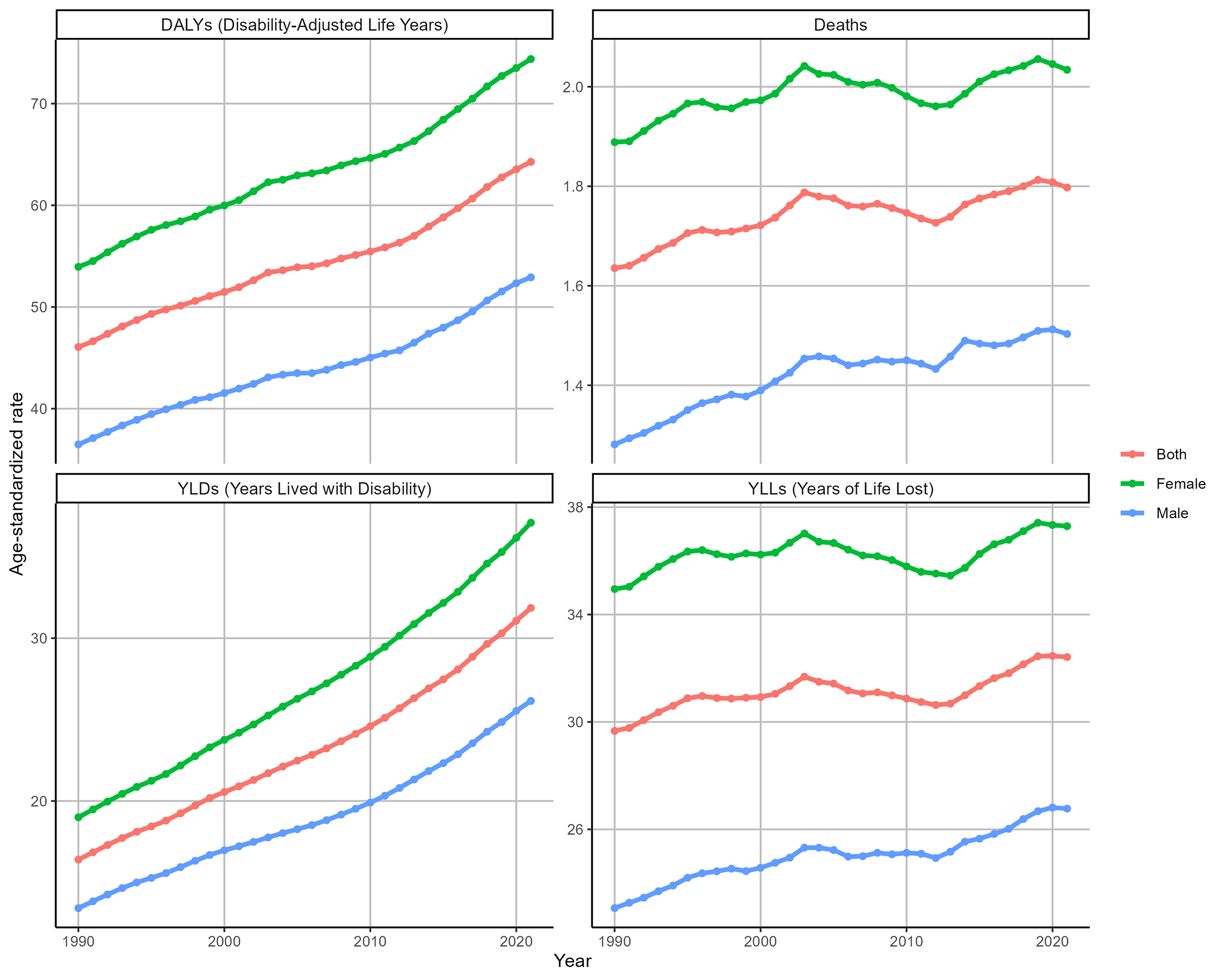

Supplement: Supplementary file 4 [file Image3.tif]

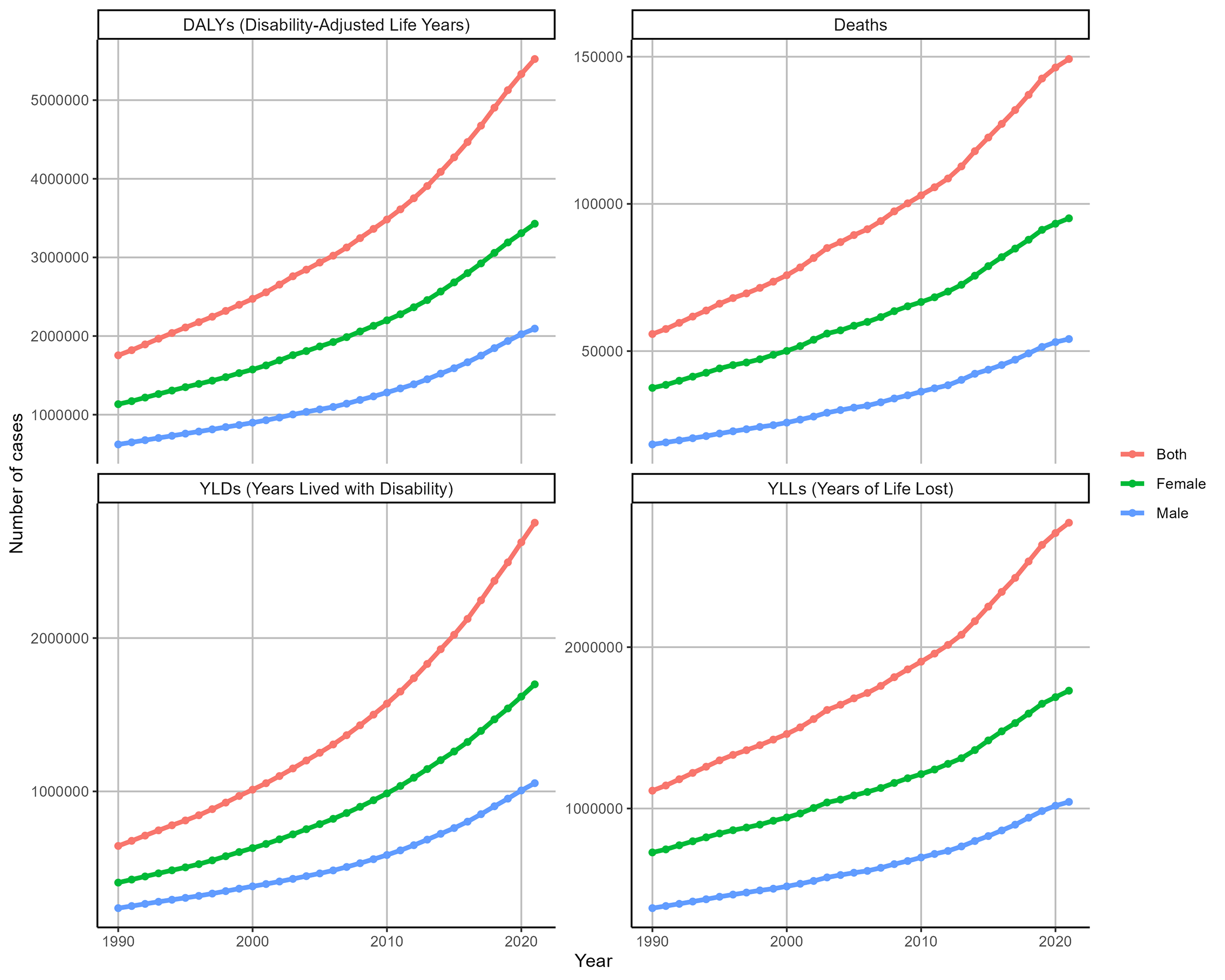

Supplement: Supplementary file 5 [file Image4.tif]

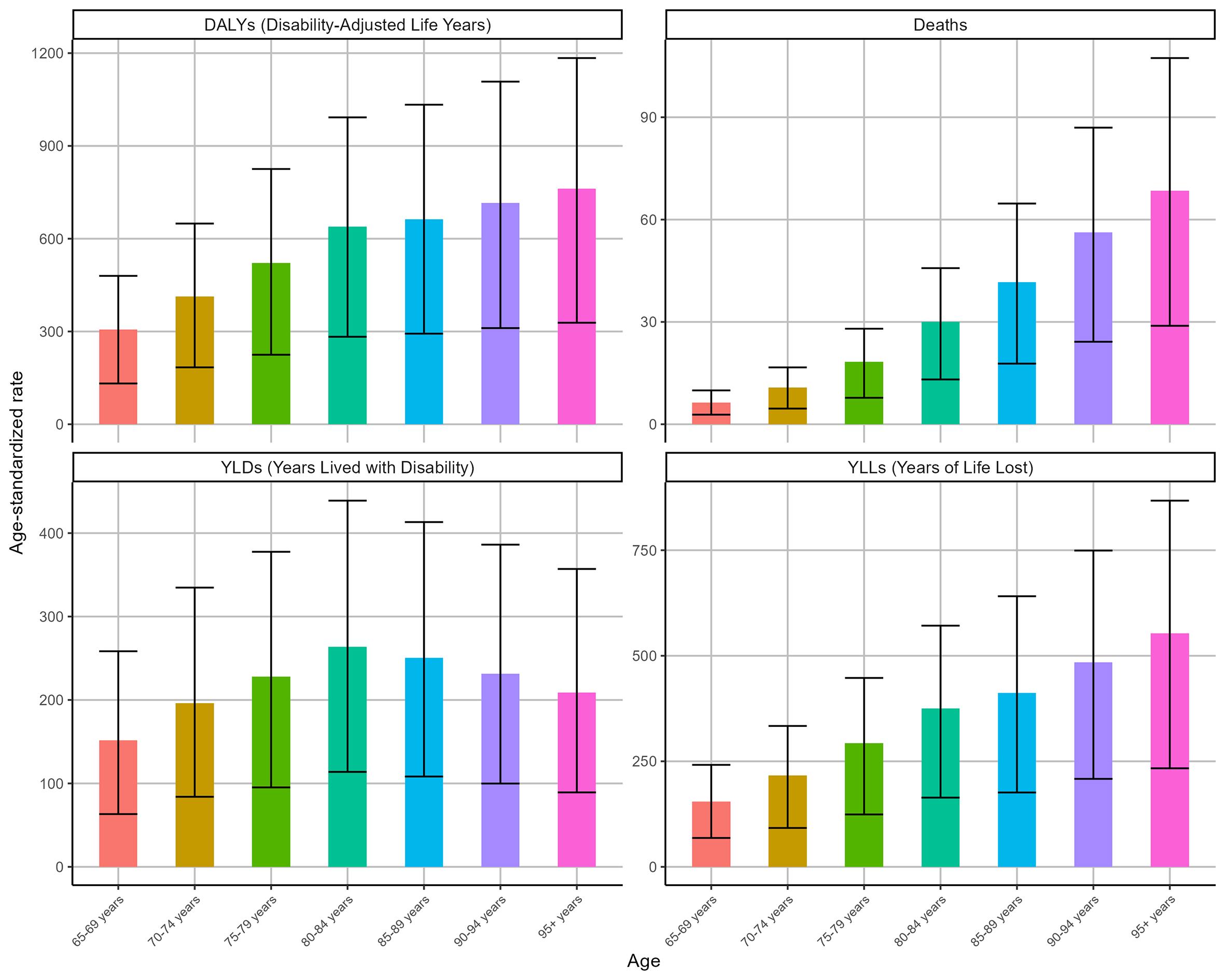

Supplement: Supplementary file 6 [file Image5.tif]

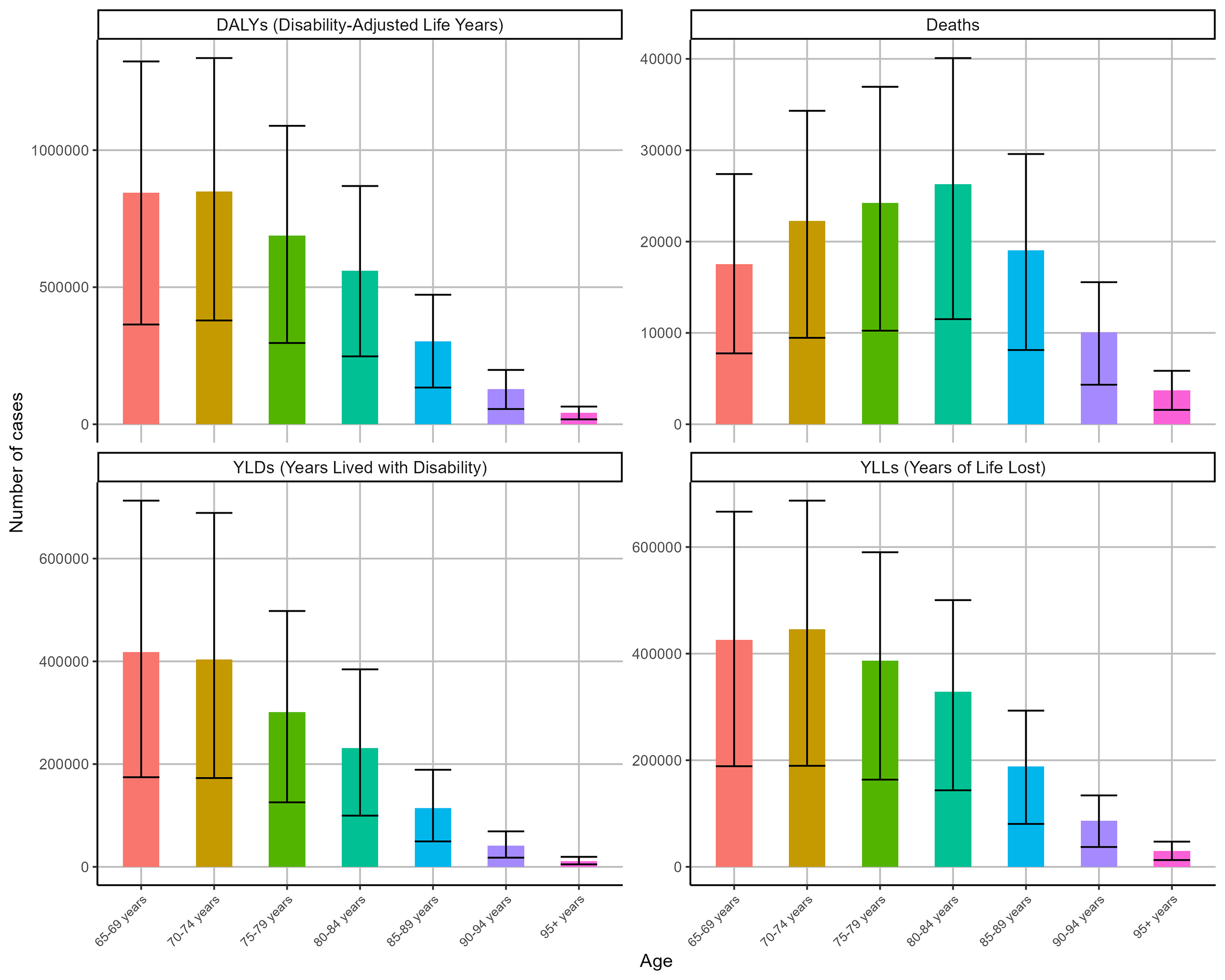

Supplement: Supplementary file 7 [file Image6.tif]

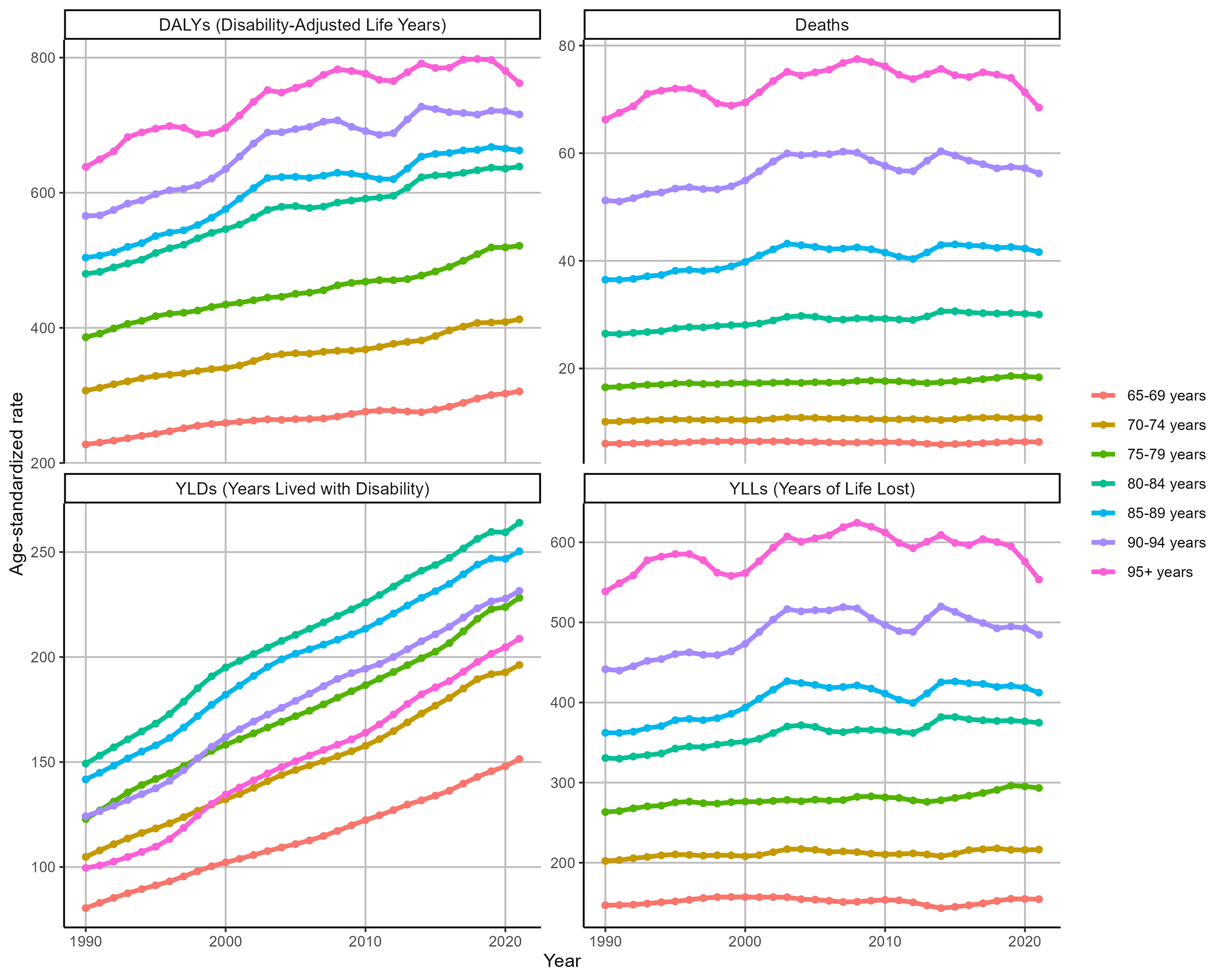

Supplement: Supplementary file 8 [file Image7.tif]

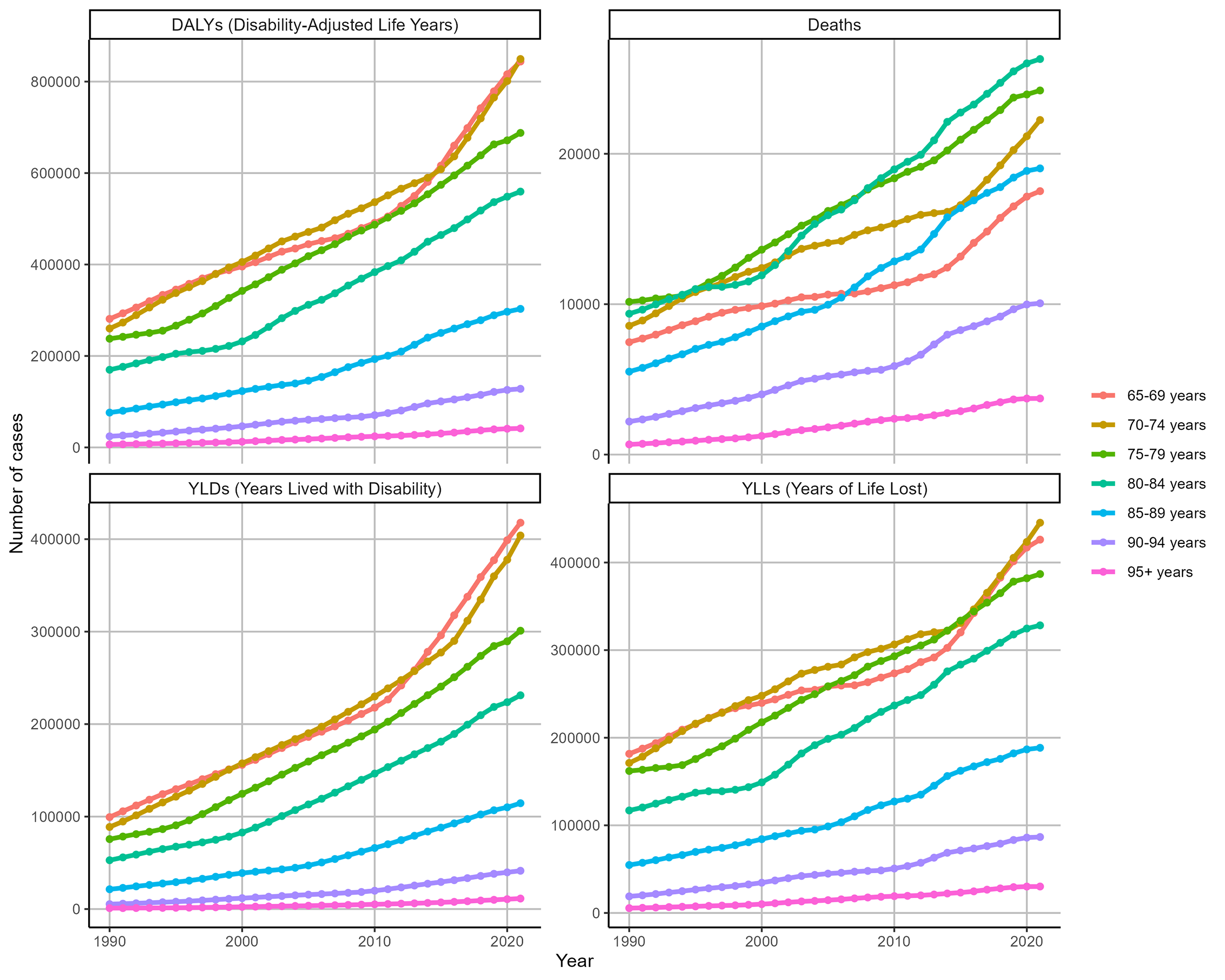

Supplement: Supplementary file 9 [file Image8.tif]

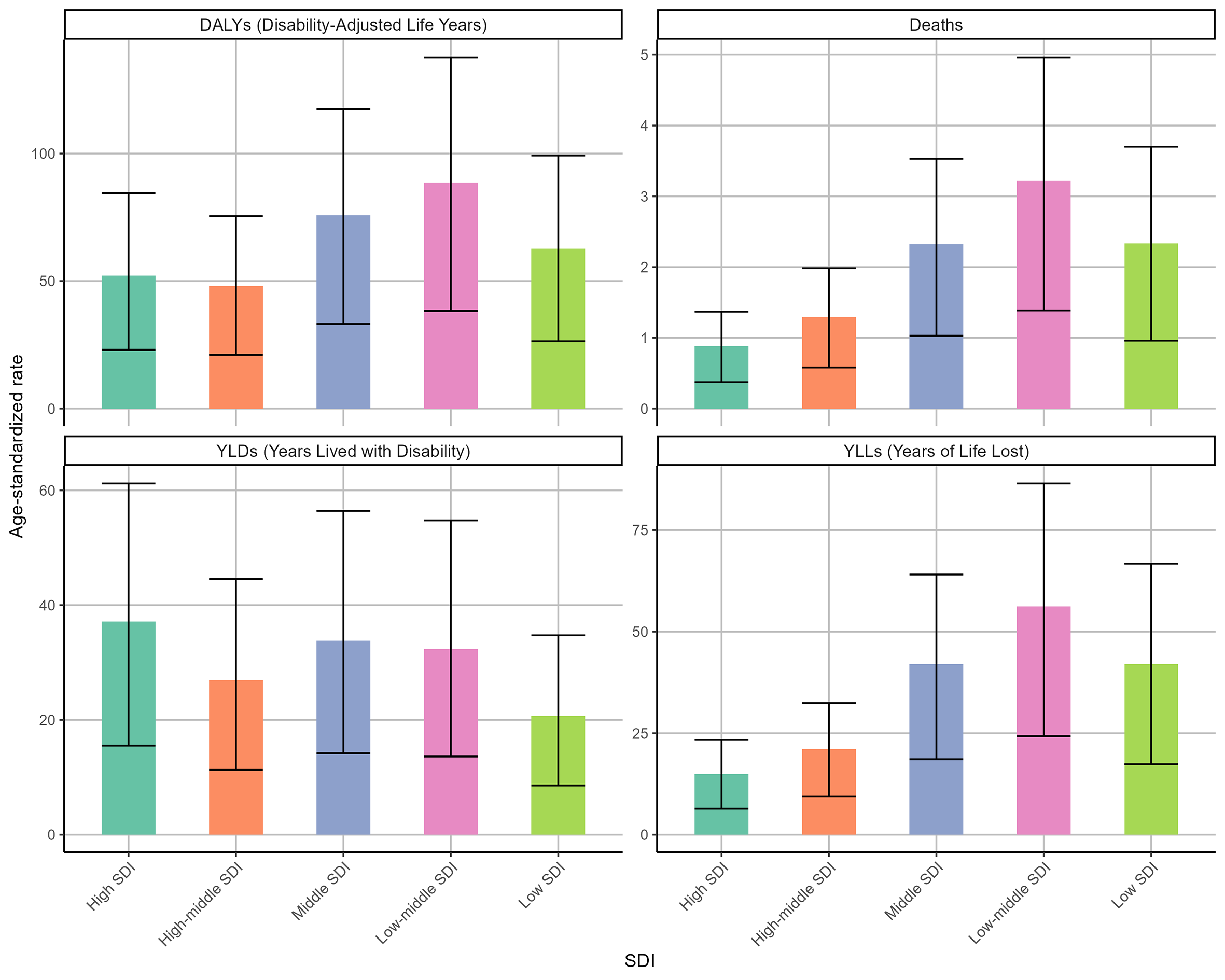

Supplement: Supplementary file 10 [file Image9.tif]

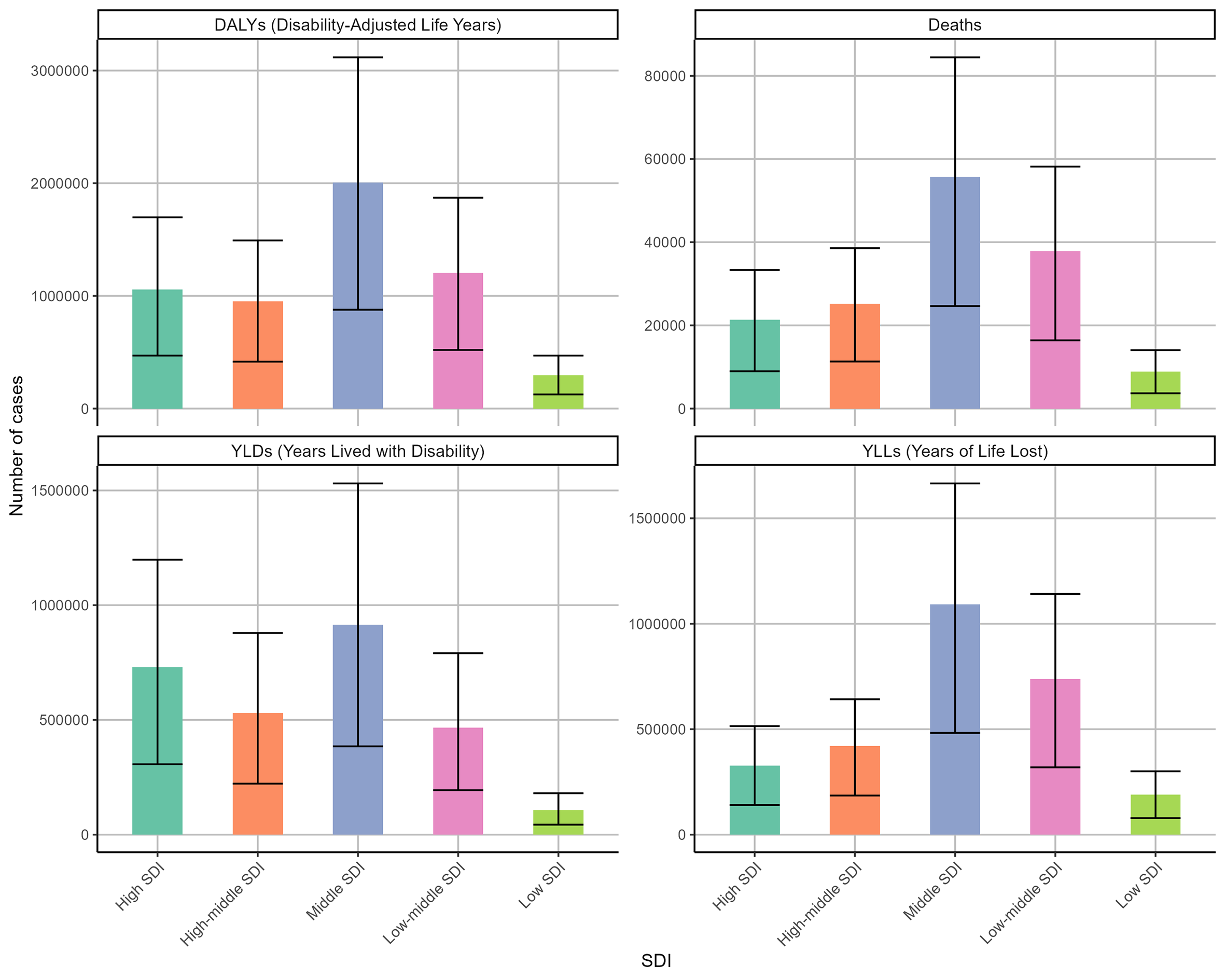

Supplement: Supplementary file 11 [file Image10.tif]

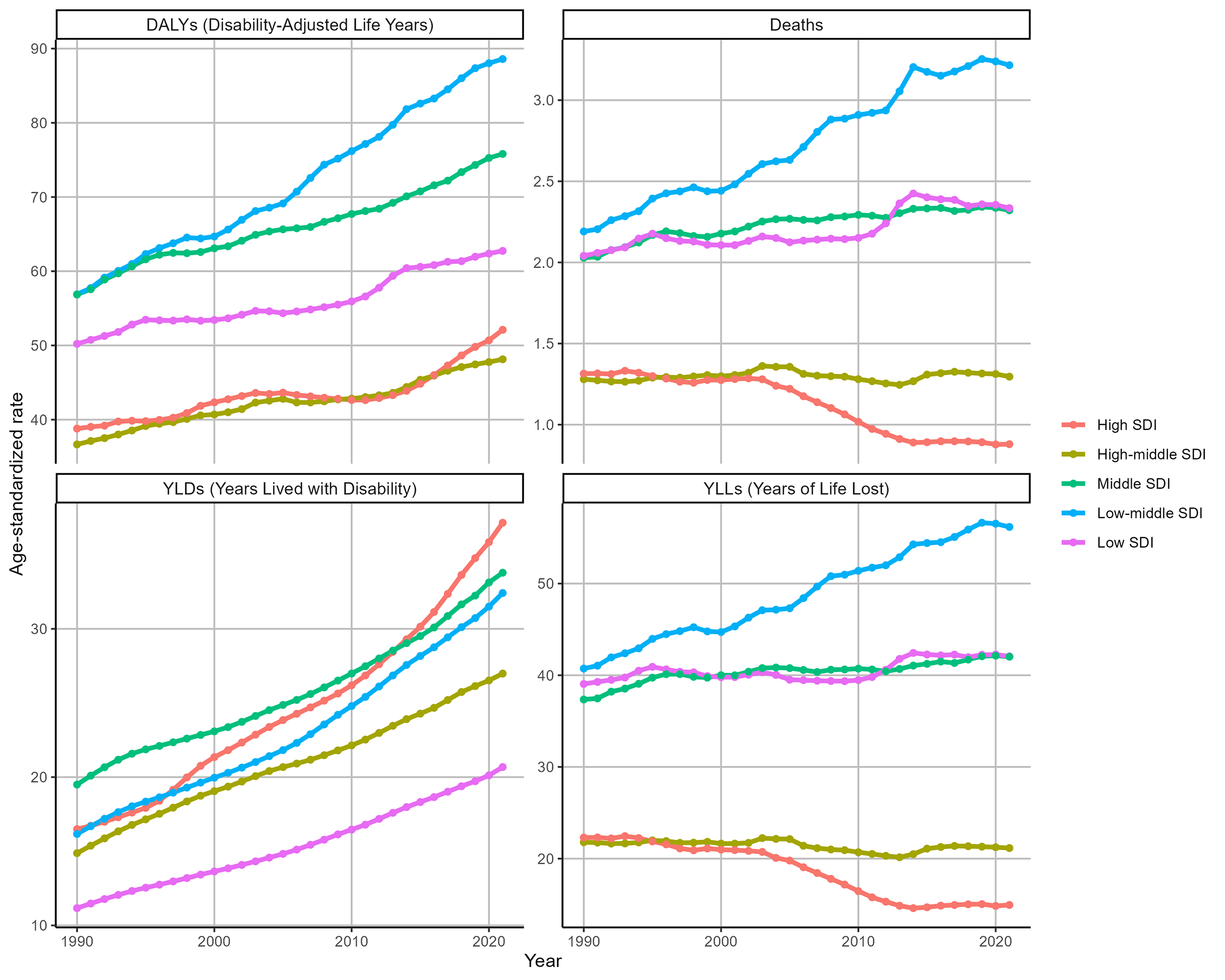

Supplement: Supplementary file 12 [file Image11.tif]

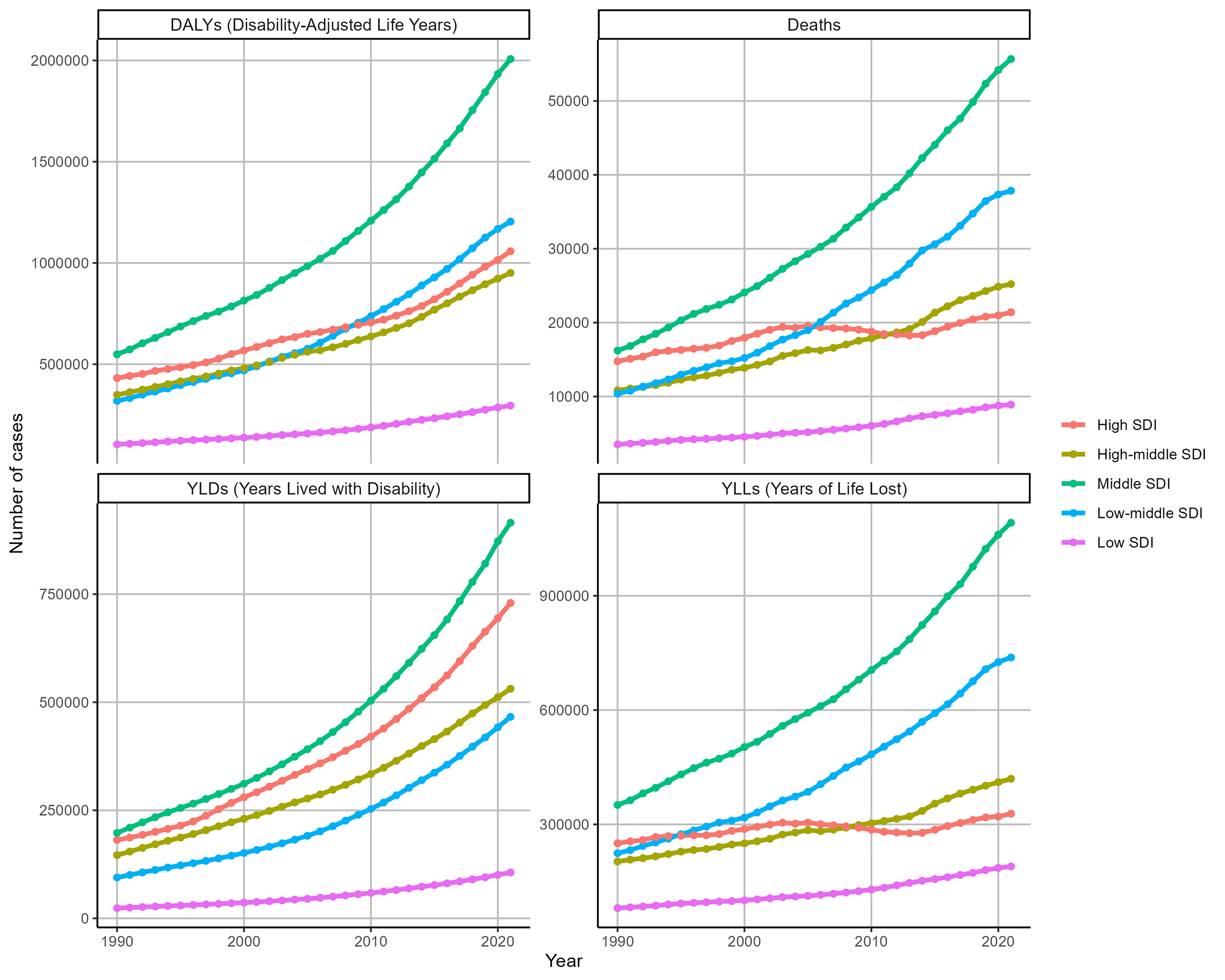

Supplement: Supplementary file 13 [file Image12.tif]

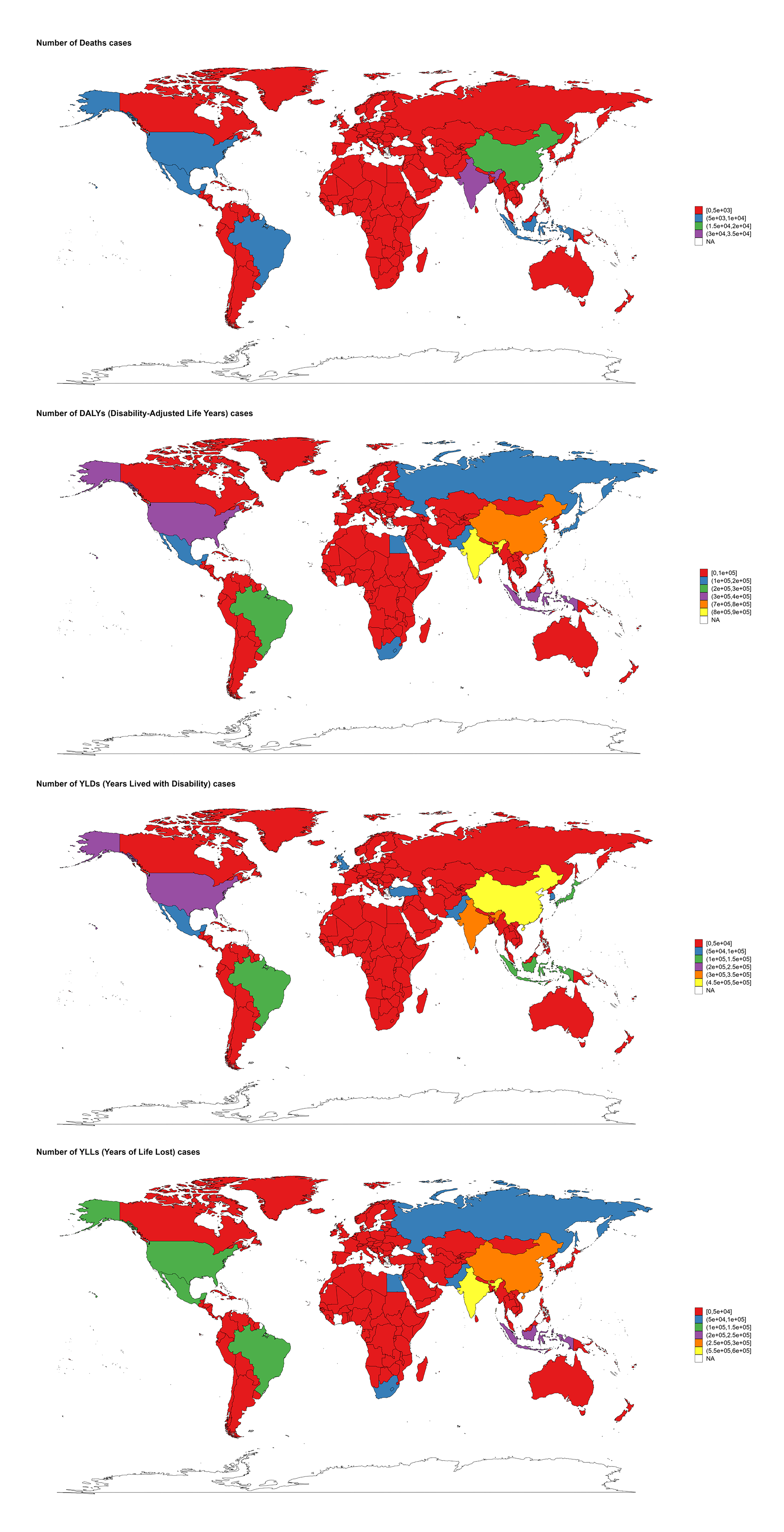

Supplement: Supplementary file 14 [file Image13.tif]
